# A RUSBoosted tree method for k-complex detection using tunable Q-factor wavelet transform and multi-domain feature extraction

**DOI:** 10.3389/fnins.2023.1108059

**Published:** 2023-03-14

**Authors:** Yabing Li, Xinglong Dong

**Affiliations:** ^1^School of Computer Science and Technology, Xi'an University of Posts and Telecommunications, Xi'an, Shaanxi, China; ^2^Shaanxi Key Laboratory of Network Data Analysis and Intelligent Processing, Xi'an University of Posts and Telecommunications, Xi'an, Shaanxi, China; ^3^Xi'an Key Laboratory of Big Data and Intelligent Computing, Xi'an University of Posts and Telecommunications, Xi'an, Shaanxi, China

**Keywords:** k-complexes detection, electroencephalogram (EEG), multi-domain features extraction, tunable-Q factor wavelet transform, RUSBoosted tree model

## Abstract

**Background:**

K-complex detection traditionally relied on expert clinicians, which is time-consuming and onerous. Various automatic k-complex detection-based machine learning methods are presented. However, these methods always suffered from imbalanced datasets, which impede the subsequent processing steps.

**New method:**

In this study, an efficient method for k-complex detection using electroencephalogram (EEG)-based multi-domain features extraction and selection method coupled with a RUSBoosted tree model is presented. EEG signals are first decomposed using a tunable Q-factor wavelet transform (TQWT). Then, multi-domain features based on TQWT are pulled out from TQWT sub-bands, and a self-adaptive feature set is obtained from a feature selection based on the consistency-based filter for the detection of k-complexes. Finally, the RUSBoosted tree model is used to perform k-complex detection.

**Results:**

Experimental outcomes manifest the efficacy of our proposed scheme in terms of the average performance of recall measure, AUC, and F_10_-score. The proposed method yields 92.41 ± 7.47%, 95.4 ± 4.32%, and 83.13 ± 8.59% for k-complex detection in Scenario 1 and also achieves similar results in Scenario 2.

**Comparison to state-of-the-art methods:**

The RUSBoosted tree model was compared with three other machine learning classifiers [i.e., linear discriminant analysis (LDA), logistic regression, and linear support vector machine (SVM)]. The performance based on the kappa coefficient, recall measure, and F_10_-score provided evidence that the proposed model surpassed other algorithms in the detection of the k-complexes, especially for the recall measure.

**Conclusion:**

In summary, the RUSBoosted tree model presents a promising performance in dealing with highly imbalanced data. It can be an effective tool for doctors and neurologists to diagnose and treat sleep disorders.

## 1. Introduction

In addition to monitoring sleep disorder disease, sleep analysis hinged on an electroencephalogram (EEG) can also play a critical role in people's mental and physical health (Al-Salman et al., 2021, 2022b). K-complex, as one of the most prominent transient waveforms in sleep stage 2, is usually utilized for sleep research and clinical diagnosis (Al-Salman et al., 2019b; Latreille et al., 2020). Due to this significance, the determination of the k-complex in an epoch is extremely important for sleep experts. K-complex, which was first discovered in Loomis et al. (1938), is a transient waveform of more than ±75 mV for a first negative sharp wave immediately followed by a slower positive component, and it was also reported that the frequency scales focus on 12–14 Hz waves (Richard and Lengellé, 1998). The duration of k-complexes was between 1 and 2 s, and other studies reported that the maximum duration is between 1 and 3 s (Al-salman et al., 2018; Al-Salman et al., 2019b). In general, k-complex detection based on sleep specialist visually scored is regarded as the gold standard. However, it is time-consuming, subjective, and onerous (Lajnef et al., 2015). Thus, more and more researchers focus on developing an automatic k-complex detection method to speed up diagnosis and alleviate the burden of neurologists.

A large number of studies on the automated detection of the k-complexes have been developed, which focus on feature extraction, feature selection, and pattern recognition stages. Some studies presented the literature concerning feature extraction, such as temporal information (Hassan and Bhuiyan, 2016a, 2017a; Al-Salman et al., 2022a), spectral estimation (Herman et al., 2008; Hassan and Subasi, 2016), and chaotic information estimation (Peker, 2016; Al-salman et al., 2018; Al-Salman et al., 2019a; Nawaz et al., 2020). Aykut et al. employed features based on amplitude and duration properties of the k-complex waveform, and the results were evaluated with the ROC analysis which proved up to 91% success in detecting the k-complex (Erdamar et al., 2012). Hassan et al. presented a method of analyzing EEG waveforms based on the spectral features computed from tunable Q-factor wavelet transform (TQWT) sub-bands, and the reported results were significantly better than the existing results (Hassan and Bhuiyan, 2016b). The scheme based on TQWT and bootstrap aggregating for EEG signals was developed, and the results showed that the proposed method is superior in terms of sensitivity, specificity, and accuracy (Hassan et al., 2016). Tokhmpash et al. used the TQWT method to transform EEG signals, and then various features were extracted from the TQWT sub-bands. The empirical results showed the high efficiency of the proposed method in the analyzing of EEG signals (Tokhmpash et al., 2021). The TQWT is also applied to decompose an EEG signal into various sub-bands at different levels; the findings showed that the proposed scheme with estimating the Hjorth parameters preserves efficiency and is appropriate for the automated identification of EEG signals (Geetika et al., 2022). Some time and frequency analysis methods based on variational mode decomposition were utilized to determine the k-complex, and the highest average accuracy was obtained at 92.29% (Yücelbaş et al., 2017). Wessam proposed an efficient method based on fractal dimension to detect k-complexes from EEG signals, and the findings revealed that the proposed method yields better classification results than other existing methods (Al-Salman et al., 2019b).

However, to the best of our knowledge, one of the state-of-the-art linear or non-linear features in the detection of k-complex has not been undertaken yet. Hence, selecting optimal feature sets plays an essential role in the k-complex detection system. In recent years, various methods have been applied successfully in many fields to realize the optimal feature subset selection (Xu et al., 2020; Jainendra et al., 2021). Moreover, pattern recognition techniques also offer a great potential to analyze EEG signals more effectively, which is typically based on supervised or unsupervised approaches (Hassan and Bhuiyan, 2017b; Zhang et al., 2022). Rakesh et al. put forward a fuzzy neural network for k-complex and achieved better results with an accuracy of 87.65% and a sensitivity of 94.04% (Ranjan et al., 2018). Ankit et al. presented a sparse optimization method, and the authors concluded that the proposed method is promising for the practical detection of k-complex (Parekh et al., 2015). Huy et al. proposed a hybrid-synergic machine learning method to detect k-complex, and the results indicate that the performance of the proposed model was at least as good as a human expert (Vu et al., 2012). The ensemble model combining a least square support vector machine, k-means, and naive Bayes is used to identify the detection of the k-complex. The results demonstrate that the proposed approach is efficient in EEG signals (Al-Salman et al., 2019b).

To build a reliable detection model, adequate volumes of k-complexes and non-k-complex datasets are necessary. Unfortunately, the number of epochs obtained from EEG signals with non–k-complexes is greater to a larger degree than that of those with k-complexes. Considering that most classifiers have a strong ability to predict instances with majority volumes while having a weak ability to predict instances belonging to the minority volumes. Hence, the problem to classify imbalanced data effectively is becoming the biggest challenge in k-complex detection.

**Figure 1:**
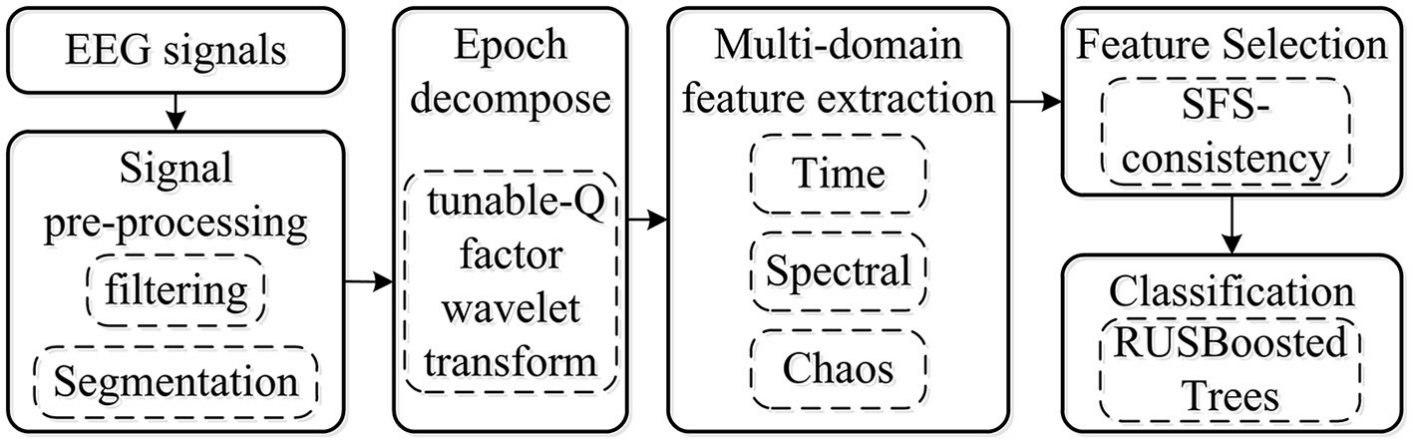
Schematic outline of the proposed computer-assisted k-complex detection scheme.

In this study, to develop and present a procedure of k-complex detection in an epoch, a robust method for the imbalance dataset was proposed based on TQWT coupled with the RUSBoosted tree classifier. The block diagram of the proposed methodology is depicted in Figure 1. Each EEG signal of 30 min was filtered with a fourth-order pass-band Butterworth filter at 0.5–30 Hz to smooth the EEG signal and remove the environment noise caused by muscle activity and eye movement. Then, the EEG signal was segmented into epochs of 0.5 s with an overlapping of 0.4 s, each epoch corresponding to a signal state for k-complex or non–k-complex. The multi-domain features (time, spectral, and chaotic theory) were extracted from each sub-bands of epoch based on TQWT decomposing. To minimize the complexity and reduce the dimensionality of features, the feature selection method based on search-based feature selection consistency (SFS consistency) is employed before classification. For further analysis, the RUSBoosted tree algorithm was implemented to improve the performance in recall for the imbalanced dataset.

## 2. Materials and methods

### 2.1. The EEG recordings

**Figure 2:**
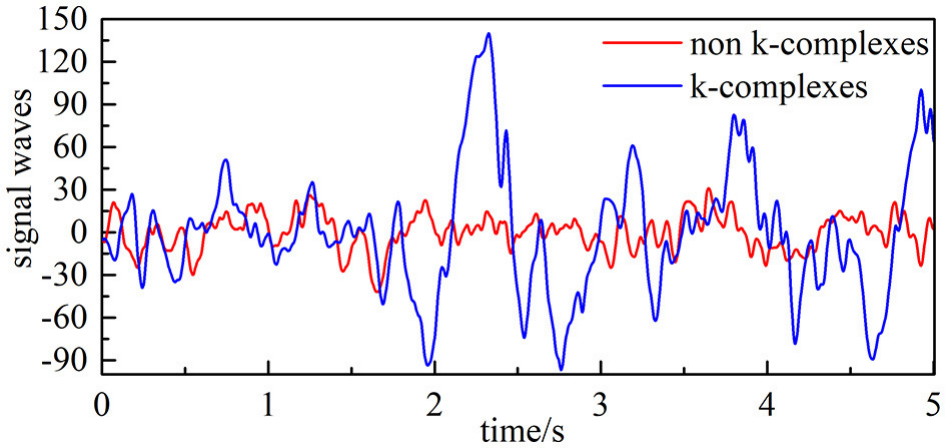
Filtered EEG signal (the blue line is EEG signals with k-complex, and the red line represents EEG signals with non–k-complex).

The EEG dataset analyzed in this study was acquired from 10 subjects (aged 28.1 ± 9.95 years, which consists of four men and six women). All were recorded at a sleep laboratory of a Belgium hospital (Brussels, Belgium) at a sampling frequency of 200 Hz, and can be found online at https://zenodo.org/record/2650142. The waveform of k-complex and non–k-complex is presented in Figure 2. The EEG recordings were visually scored by two experts with the specified recommendation (Devuyst et al., 2010). As the duration time of the k-complex is about 0.5–2 s, the EEG signals were divided into segments for k-complex detection using the sliding window technique (Siuly et al., 2011; Al-Salman et al., 2021). Based on previous empirically-based studies, the window size was selected as 0.5 s with an overlap of 0.4 s in this study (Al-Salman et al., 2019c). The multi-domain features based on the analysis of the EEG signals were employed to represent k-complex and non–k-complex from each 0.5 s EEG segment. All the analyses were carried out based on the Cz-A1 channel.

For the DREAMS database, only five of the 10 subjects are annotated by two experts, and the rest are annotated by expert 1. In this study, two different evaluation scenarios were used. The first scenario considers the annotations marked by expert 1 for all subjects, and the second scenario consists of the annotations marked by expert 2 for the five subjects. Table 1 presents the number of k-complex by the experts for Scenarios 1 and 2 in the DREAMS database. It is found that the number of k-complex by the first expert is dramatically greater than the number by the second expert. Therefore, the choice of different scenarios has a direct influence on the results and can be used to verify the performance of the proposed method.

**Table 1:** Number of k-complex in each EEG recording.

**Subject**	**Scenario 1**		**Scenario 2**	
	**Number of segments with k-complex**	**Number of segments without k-complex**	**Number of segments with k-complex**	**Number of segments without k-complex**
ID1	263	17,733	95	17,901
ID2	299	17,697	41	17,955
ID3	104	17,892	14	17,982
ID4	661	17,335	60	17,936
ID5	285	17,711	98	17,898
ID6	204	17,792	/	/
ID7	87	17,909	/	/
ID8	36	17,960	/	/
ID9	26	17,970	/	/
ID10	117	17,879	/	/

### 2.2. Tunable Q-factor wavelet transform (TQWT)

**Figure 3:**
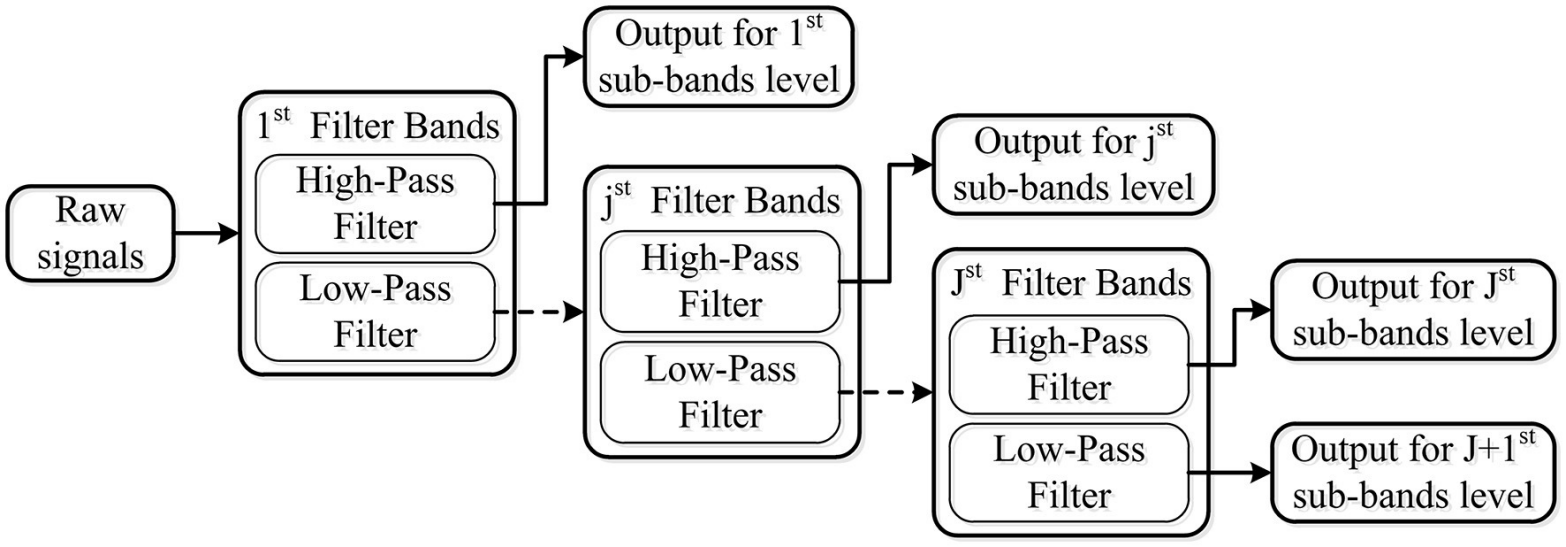
Wavelet transform with J level using a two-channel filter bank, which consists of the low-pass filter and high-pass filter.

The tunable Q-factor wavelet transform, which is proposed by Selesnick (2011), is a flexible discrete wavelet transform (DWT). Similar to the DWT, TQWT employs a two-channel filter bank, which consists of a low-pass filter with parameter α and a high-pass filter with parameter β, to decompose EEG signal into transient components and sustained components using adjustable Q-factors. It can be expressed mathematically as Equations 1, 2. For further analysis, the sustained component's output of the low-pass filter is regarded as the input signal for the next two-channel filter bank. The transient components' output of the high-pass filter for each layer is deemed as the output signal. One simple example of wavelet transform with J level is illustrated in Figure 3.


(1) $$H_{L}^{J}=\{\begin{array}{cc} \prod_{j=0}^{J-1}H_{L}\left(\omega /\alpha^{j}\right) & \left|\omega \right|\leq \alpha^{J}\pi \\ 0 & \alpha^{J}\pi \leq \left|\omega \right|\leq \pi \end{array}$$

(2) $$\begin{array}{l} H_{H}^{J}\\ =\{\begin{array}{cc} H_{H}\left(\omega /\alpha^{J-1}\right)\prod_{j=0}^{J-2}H_{L}\left(\omega /\alpha^{j}\right) & \left(1-\beta \right)\alpha^{J-1}\pi \leq \left|\omega \right|\leq \alpha^{J-1}\pi \\ 0 & others \end{array} \end{array}$$

Here,


(3) $$\begin{array}{c} \begin{array}{c} H_{L}=\theta \left(\frac{\omega +\left(\beta -1\right)\pi }{\alpha +\beta -1}\right)\\ H_{H}=\theta \left(\frac{\alpha \pi -\omega }{\alpha +\beta -1}\right)\\ \theta \left(t\right)=0.5\left(1+\cos\left(t\right)\right)\sqrt{2-\cos\left(t\right)} \end{array} \end{array}$$

Q-factor: This parameter determines the width of the band-pass filter. TQWT decomposition achieves flexibility by tuning and adapting this parameter of the wavelet transform. The higher the Q-factor is, the more effective the extraction of the sustained components. Meanwhile, the decomposing waveform based on a lower Q-factor is suitable for extracting the features of the transient component.

Number of decomposition levels (J): If the number of filter bands is denoted by J, an input signal will be decomposed into J+1 sub-bands. Among these bands, J sub-bands were obtained from the high-pass filter of each level filter band, and one came from the low-pass filter of the final level filter band. With the increase of the decomposition level, the time domain waveform becomes wider, and the features increase dramatically.

Taking into consideration various ranges of motivation, the TQWT is used in the proposed scheme (Hassan and Bhuiyan, 2016b). First of all, considering that k-complex waves are characterized by the appearance of multifarious rhythms, TQWT can improve localization in the frequency domain by varying the Q-factor. Hence, this decomposition method is suitable for spectral analysis. Second, the filters employed in TQWT are more computationally efficient in the frequency domain (Selesnick, 2011). Third, EEG is a non-stationary signal and its chaos properties alter between k-complex and non–k-complex. TQWT decomposition can also give the wave in the time domain; hence, it has emerged as a powerful technique in both time features and chaos features for EEG analysis (Fraiwan et al., 2010). These superiorities verified that the TQWT decomposition is an effective tool for the analysis of EEG and hence it is employed in the proposed scheme.

### 2.3. Multi-domain feature extraction from TQWT sub-bands

To derive salient features from the raw EEG data that can effectively reflect the epochs to the respective k-complex is the main objective of the feature extraction stage of the EEG-based k-complex detection system. Hence, a multi-domain method, based on time domain estimation, spectral estimation, and chaotic analysis, was employed to extract the representative features from each 5 s EEG epoch. A total of 25 hybrid features were extracted from each sub-band.

**Figure 4:**
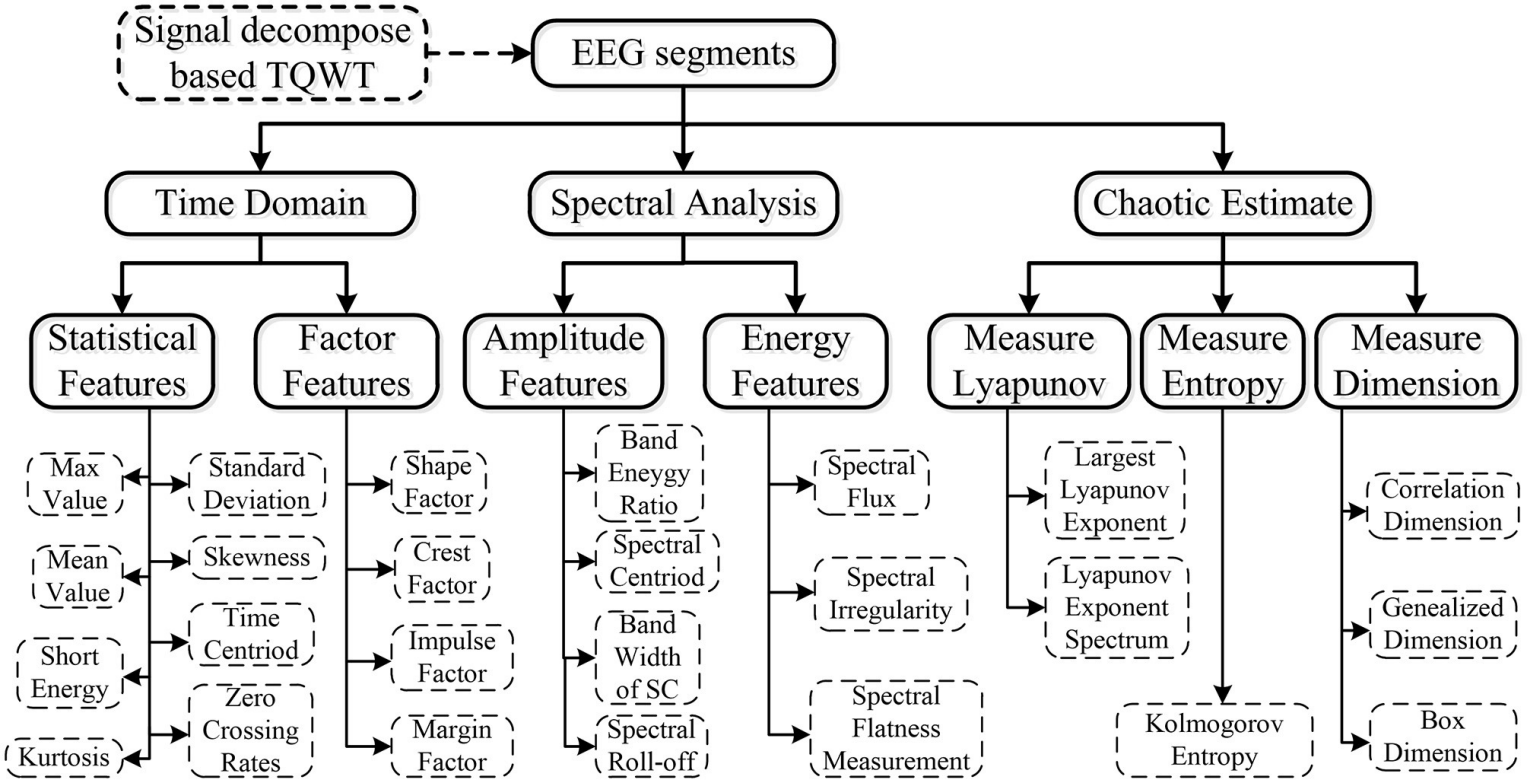
Multi-domain features extraction framework.

The extraction feature methods based on the time domain have been proven to be an efficient method for analyzing the characteristics of EEG signals (Vidaurre et al., 2009). Though it is widely used in speech and audio signal classification (Chu et al., 2009), spectral features have been used for EEG signals (Hassan and Bhuiyan, 2016b). These features are typically calculated by applying a fast Fourier transform (FFT) to short-time window segments of EEG signals followed by further processing. Considering that the property of EEG signals is somewhat chaotic, in addition to the traditional features of the EEG signal, the chaotic features based on non-linear dynamical analysis are also highly recommended to investigate the dynamic characteristics of EEG (Li et al., 2017; Nawaz et al., 2020). In the current study, 12 time domain features, seven spectral features, and six chaotic features are extracted for further analysis, as shown in Figure 4.

We have computed the feature vector for each EEG sub-bands based on TQWT decomposition. As the decomposed EEG signals with J+1 sub-bands, the feature vector of J+1 sub-bands on each epoch is computed to construct a 25^*^(J+1)-dimensional feature vector.

### 2.4. Search-based feature selection using consistency measures

Considering that reducing the dimensionality of feature sets may be improving the performance in reducing costs and enhancing the ability of comprehensibility, another effective step in the detection system for k-complex is to find optimal feature subsets. Selection features based on search-based feature selection (SFS) analyses were used in this study to research and select the important features. The following context briefly illustrates the selection features (Dash and Liu, 2003; Hernández-Pereira et al., 2016).

**Figure 5:**
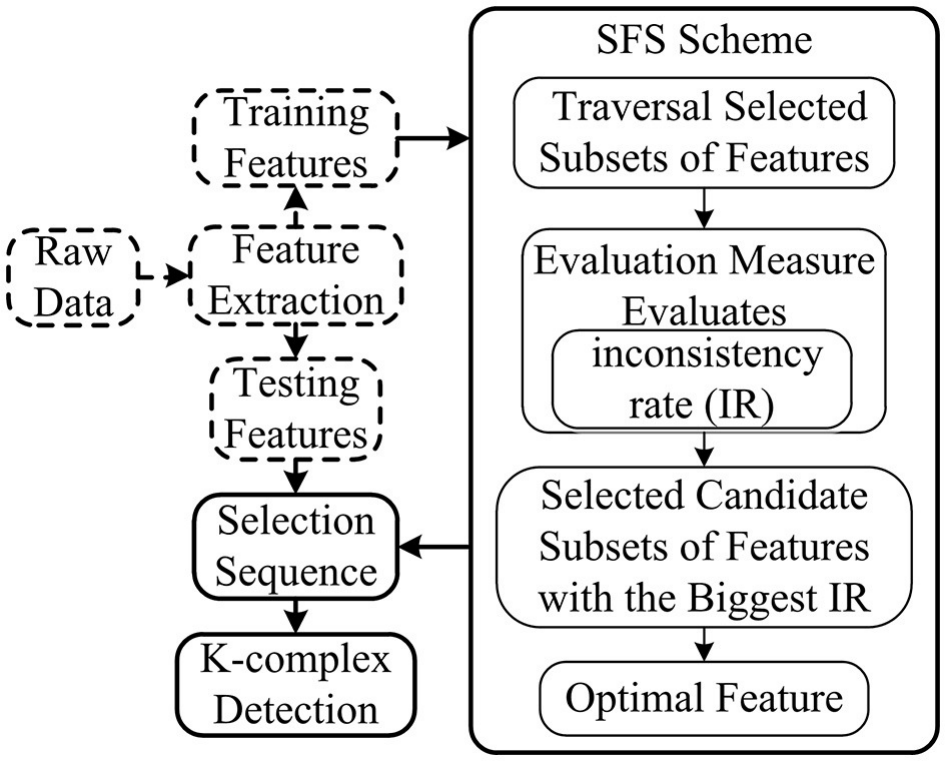
Scheme of SFS. The dimension of feature subsets is reduced based on features selected by SFS, and the selected features are used for further analysis.

The SFS method based on the consistency filter, as one of the most effective methods, traverses all the candidate subsets to find the best one using the evaluation measures based on the independence of an inductive algorithm (shown in Figure 5). The evaluation measure evaluates the attributes of selected features according to the inconsistency rate (IR). If the IR for current selection features is smaller than the pre-selection features, current selection features are deemed as the selected features. Although SFS has the disadvantage in time-consuming, it does not need the stopping criterion or a pre-specified threshold.

### 2.5. RUSBoosted tree model for the k-complex detection

The distribution across k-complex or not is highly skewed: non–k-complexes have more epochs than those k-complexes. Therefore, the detection problem for the imbalanced dataset is a major challenge for k-complex detection. The RUSBoosted tree model, as an efficient way to overcome this problem, can improve the prediction performance by reducing bias between positive and negative samples at the expense of a slight decrease in the large group sets (Khoshnevis and Sankar, 2020; Jain and Ganesan, 2021; Noor et al., 2022).

**Figure 6:**
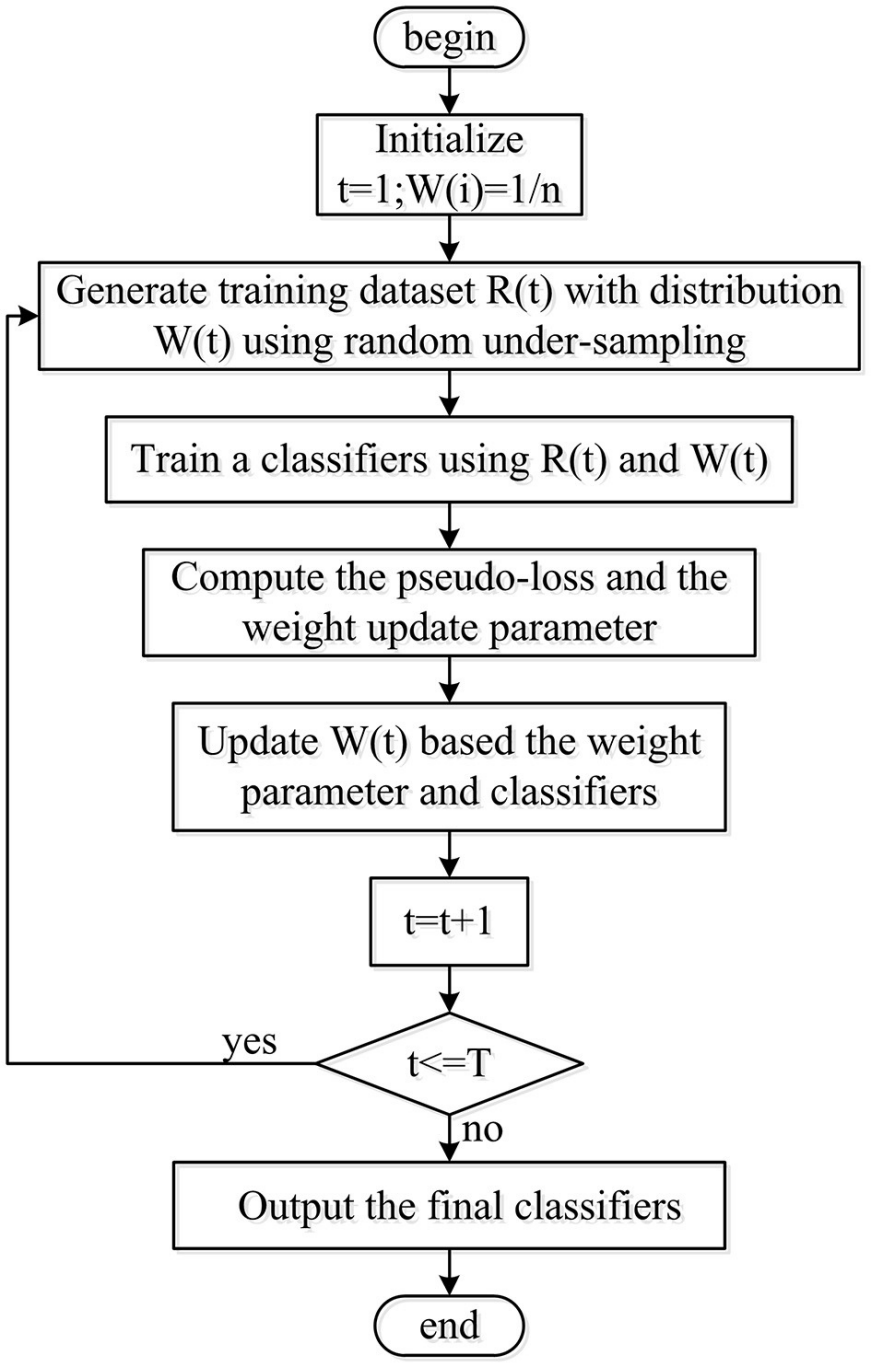
Flowchart for the RUSBoost implementation.

The present research fused a random under-sampling (RUS) technique and adaptive boosting (AdaBoost) algorithm with a decision tree as the RUSBoosted tree model, as shown in Figure 6. First of all, to obtain the balanced distribution, the under-sampling method was implemented to deal with the minority and majority class size for the imbalanced training dataset. Second, considering the AdaBoost algorithm's ability to reduce bias and variance mistakes, it is employed to tackle problems involving imbalanced datasets. Hence, the RUS technique along with AdaBoost is utilized by combining an ensemble of decision trees as a classifier for further analysis.

In this study, the parameters (i.e., the number of classifiers was selected as 30 for the model, with a maximum number of splits of 20 and a learning rate of 0.1) were melded into the RUSBoosted tree for the detection of k-complex.

### 2.6. Performance evaluation

First, statistical hypothesis testing is performed to validate the relevance and suitability of features according to discriminatory capability are statistically significant or not. If the features are not statistically significant, they have to be ignored for negative influence on performance. To estimate the significant level of k-complexes and non–k-complexes, we perform a one-way analysis of variance (ANOVA). The difference is considered to be statistically significant if the *p*-value is < 0.05 at a 95% confidence level.

Second, to evaluate the detection ability of the proposed method, some metrics based on the confusion matrix (shown in Table 2) were used. In Table 2, TP describes the situation that both the actual k-complexes and predicted states are yes. FN represent the situation that predicted k-complexes as no while actual k-complexes as yes. FP means the actual state is not k-complexes, which is adverse to the predicted label based on an algorithm. TN means the situation that both the actual k-complexes and predicted states are no.

**Table 2:** Confusion matrix of the k-complex detection problem.

		**Predicted k-complexes**	
		**Yes**	**No**
Actual k-complexes	Yes	True positive (TP)	False negative (FN)
	No	False positive (FP)	True negative (TN)

To evaluate the performance of the detection algorithm, Cohen's kappa coefficient, recall, and F-measure are computed. In addition to these metrics, the area under the ROC curve (AUC) was also used to estimate the performance of a classifier. Further details about the metrics are provided in the following paragraphs.

The kappa coefficient, calculated based confusion matrix, as a measurement for consistency tests, can also be used to measure classification accuracy. It is defined as Equation 4 as follows:


(4) $$\begin{array}{l} kappa=\frac{\frac{TP+TN}{TP+FN+FP+TN}-P_{e}}{1-P_{e}} \end{array}$$

Here, *P*_*e*_ is obtained as follows:


(5) $$P_{e}=\frac{\sum_{i}sum(M(i,:))\times sum(M(:,i))}{{\left(\sum M\right)}^{2}};M\;is\;confusion\;matrix$$

Recall measure, which is also called sensitivity measurement, reflects the proportion of the actual positive prediction. It can be expressed mathematically from Equation 6 as follows:


(6) $$\begin{array}{l} \text{Recall}=\frac{TP}{TP+FN} \end{array}$$

F-measure is the top priority measurement in analyzing the overlapping between the two sets. It can be defined by weighted recall and precision, and β reflects the relative importance.


(7) $$\begin{array}{l} F_{\beta }=\frac{\left(1+\beta^{2}\right)\times Precision\times Recall}{\left(\beta^{2}\times Precision\right)+Recall} \end{array}$$

If the parameter of β>1, it means that recall has more influence on F-measure. 0 < β < 1 reflects that precision has a broader effect on F-measure, compared with recall. β = 1 represents the measurement degenerates into standard F-measure. It is noted that β = 10 is selected.

To further illustrate the effectiveness of features selected using a feature selection-based consistency-based filter, the separability analysis using Fisher criteria was applied, which is obtained from Equation 9 as follows:


(8) $$\begin{array}{l} {\text{J}}_{\text{F}}=\text{tr}\left({\text{S}}_{\text{w}}^{-1}{\text{S}}_{\text{m}}\right) \end{array}$$

Here, *S*_*w*_ and *S*_*m*_ represent the within-class and between-class scatter matrix, respectively. tr(S) means the trace of square matrix S.

To evaluate the performance of the proposed method, the 5-fold cross-validation method is utilized. The k-complex segments and non–k-complex segments are divided into five groups, respectively. For each time, the training dataset consists of four k-complex groups and four non–k-complex, while the resting groups are deemed as testing groups. All groups are tested in turn. In this study, the overall performance is computed over the five iterations.

## 3. Results and discussion

### 3.1. Parameter selection for TQWT

**Figure 7:**
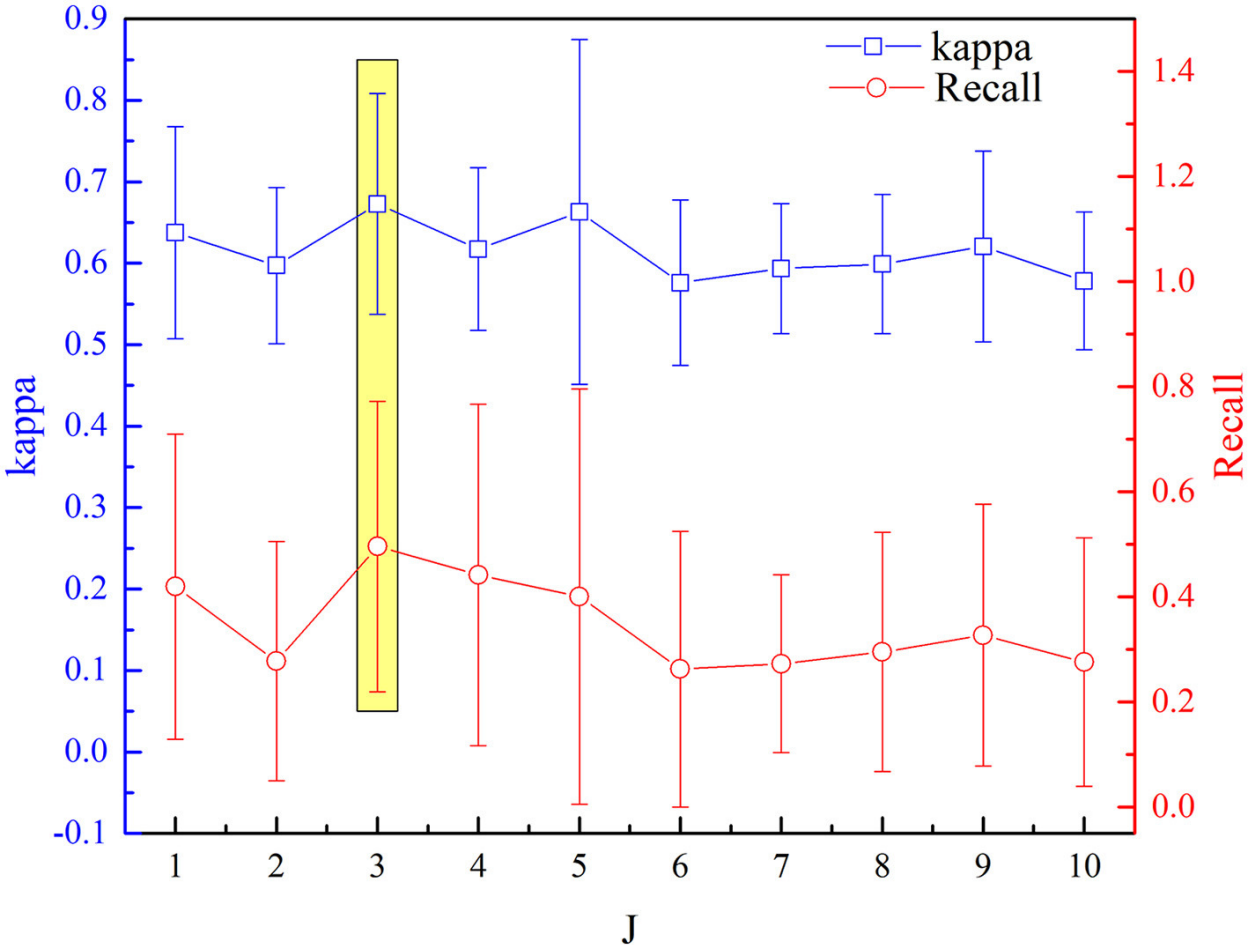
Variation of kappa and recall value with J for the detection of k-complexes.

**Figure 8:**
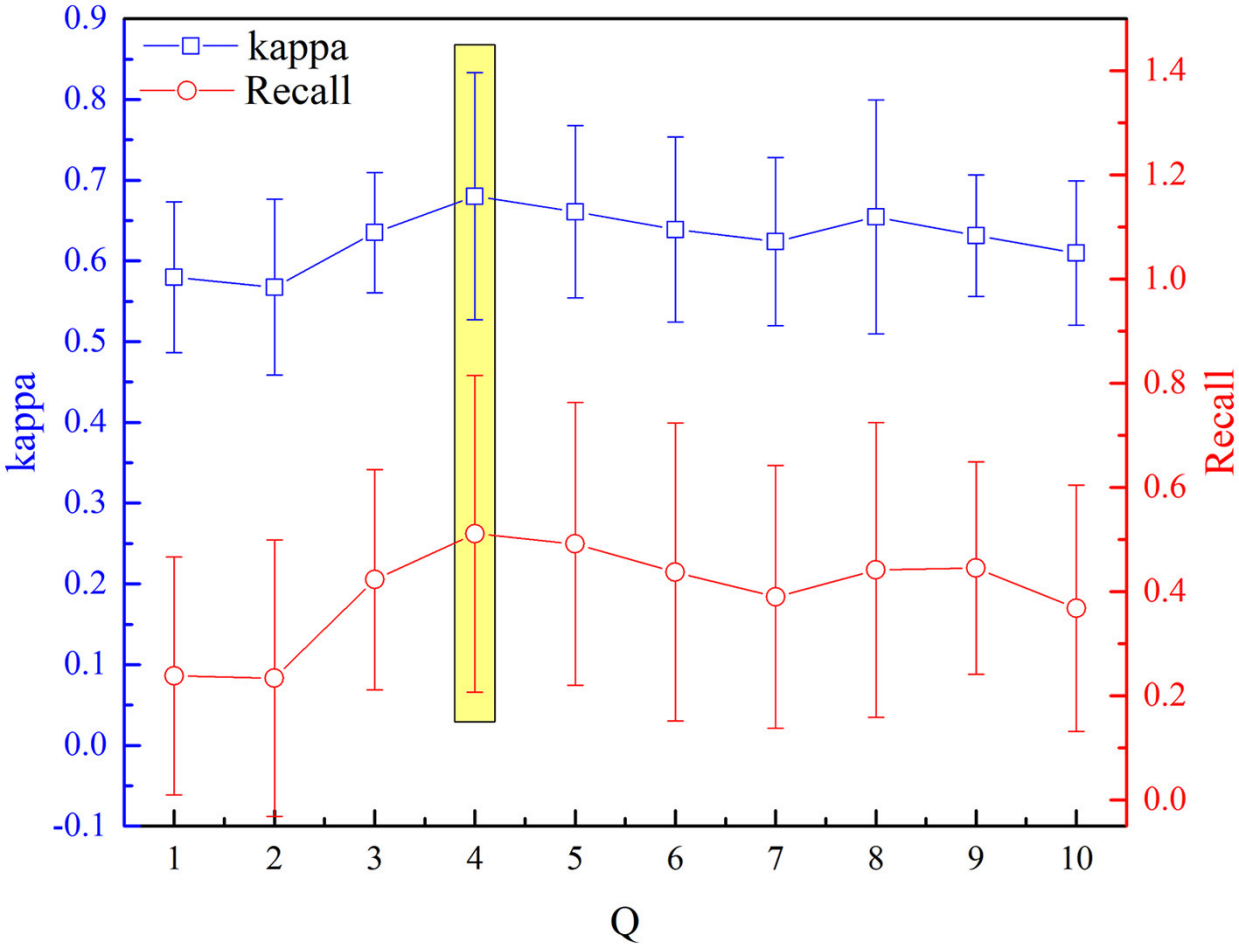
Variation of kappa and recall value with Q for the detection of k-complexes.

The selected optimal parameters to decompose the EEG epoch are J and Q. The detection performance (kappa measures and recall value) based on the aforementioned procedure of feature extraction and selection has been analyzed sequentially for incremental values of Q range from 1 to 10 with an increment of one. Figures 7, 8 depict the influence of parameters on detection performance for the k-complex. It is observed from Figure 7 that the optimal parameter of J is 3, in which the best kappa measures and recall value are achieved. The optimal value for J is determined in the same way. From our experimental analyses, as shown in Figure 8, it has been observed that the best matrices are achieved for Q = 4.

### 3.2. Quality evaluation for feature extraction and selection

In this section, the results of all the features computed from various TQWT sub-bands were present in terms of significance, as shown in Table 3. The test is performed at a 95% confidence level. It can be observed from Table 3 that the features highlighted in bold are not significant (*p* > 0.05), and a difference is statistically significant if *p* ≤ 0.05. The results show that the performance of time domain features to classify k-complex was significantly better than other features for sub-bands 1 and 2. In sub-band 3, spectral features significantly outperformed time and chaotic features. However, the statistical performance of time features in sub-band 4 was the worst in all three kinds of features. Based on these results, we can conclude that not all of the sub-bands features achieved good discriminatory capability for k-complex detection. Hence, it is necessary to select some of these features to improve the k-complex detection performance and decrease time consumption.

**Table 3:** The *P*-value of the proposed features computed from various TQWT sub-bands indicates the difference in features between k-complex and non–k-complex.

**Features**		**Sub-band 1**	**Sub-band 2**	**Sub-band 3**	**Sub-band 4**
Time features	Maximum	0.023802	0.000803	**NaN**	**0.87868**
	Mean	**0.291217**	**0.702964**	**0.308926**	**0.458965**
	Standard deviation	0.010885	0.007884	**0.546545**	**0.861432**
	Skewness	0.008726	0.000836	**NaN**	**0.881254**
	Kurtosis	**0.05654**	**0.72175**	0.048551	**0.731241**
	Shape factor	0.001473	0.008128	**0.548529**	**0.816636**
	Crest factor	0.00672	0.000878	**NaN**	**0.916789**
	Impulse factor	0.037651	**0.092862**	0.01697	**0.206266**
	Margin factor	0.000359	0.008451	0.031723	**0.683969**
	Short energy	9.47E-17	2.54E-16	**0.279627**	0.01927
	Zero-crossing rate	1.68E-13	4.13E-27	2.52E-10	9.76E-19
	Time centroid	6.49E-22	1.36E-08	5.27E-05	**0.951018**
Spectral features	Band energy ratio	**0.956247**	0.007944	**0.889128**	**0.289102**
	Spectral flux	0.004931	**0.733733**	**0.797405**	**0.780187**
	Spectral centroid	0.008618	**6.77E-01**	**0.793363**	0.002373
	Band width of SC	**0.709624**	**0.946816**	**0.363672**	0.023152
	Spectral flatness measurement	**0.561545**	**0.701588**	**0.077005**	**0.263733**
	Spectral roll-off	**0.594201**	8.66E-05	7.65E-05	2.65E-11
	Spectral irregularity	0.01874	**0.127941**	0.000109	5.51E-07
Chaotic features	Correlation dimension	**0.087149**	**6.52E-01**	**0.395171**	0.000795
	Kolmogorov entropy	**0.346332**	**0.309228**	**0.309228**	**0.865964**
	Largest Lyapunov exponent	**7.69E-01**	0.007019	0.031744	**0.890812**
	Lyapunov exponent spectrum	**0.318448**	**0.318448**	**0.813963**	**0.988225**
	Box dimension	**0.803216**	1.88E-09	0.00112	2.02E-17
	Generalized dimension	1.51E-09	2.39E-13	7.73E-24	7.73E-24

It is noted that the features with not statistically significant are highlighted in bold.

**Figure 9:**
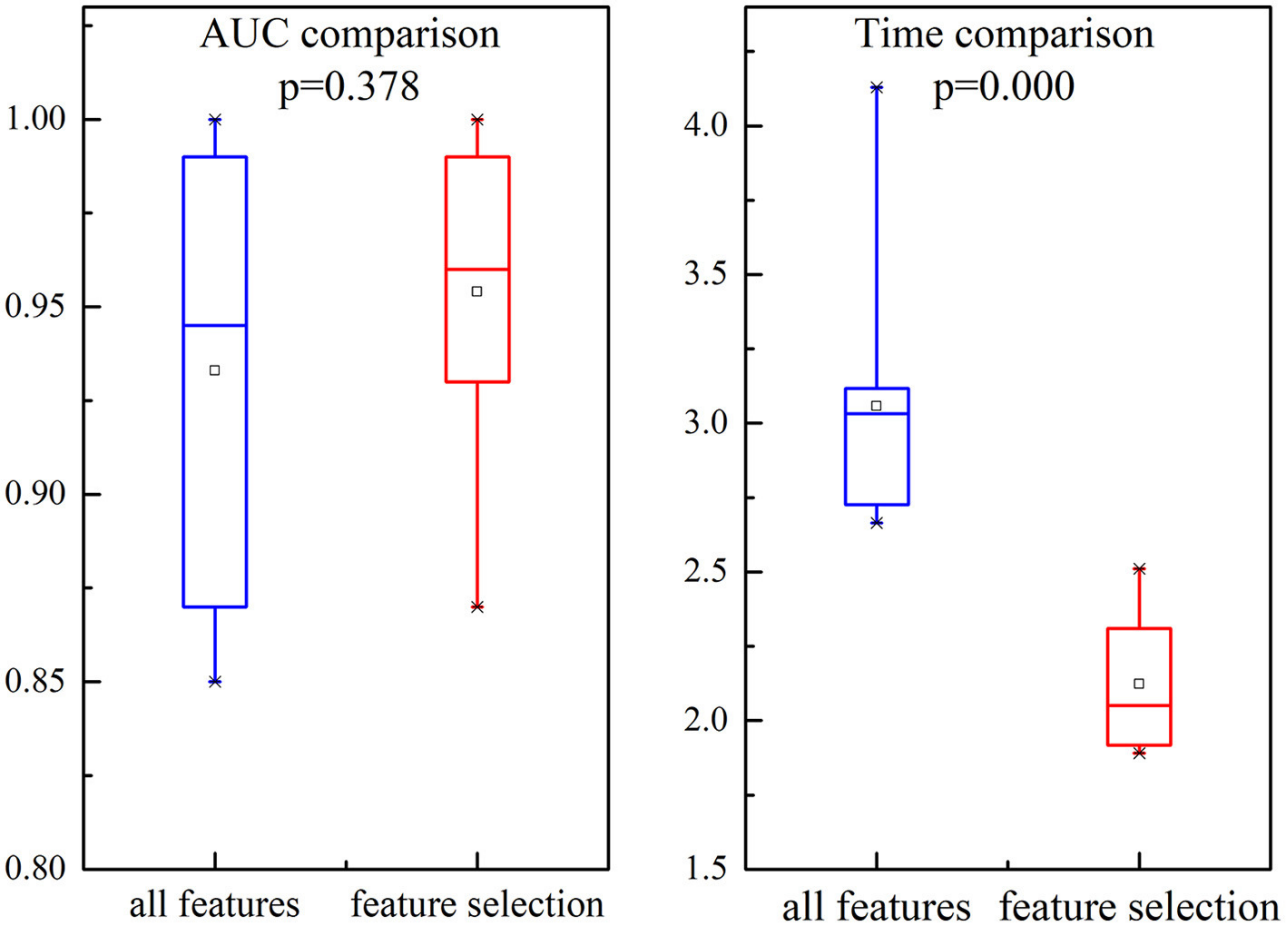
Illustration of the comparison for AUC and time in all features and selected features. Each box represents the 25–75th percentiles, the central line is the median value, and the tiny vertical lines extend to the most extreme data not considered outliers, which are plotted individually.

We investigate the AUC and time performance for two different feature sets, namely all features and selected features. The comparisons of the performance are shown in Figure 9. It is evident that the AUC based on selection features is slightly incremented than all feature sets. Compared with the performance of all feature sets, there is a dramatic decrement in time comparison for selected feature sets.

**Figure 10:**
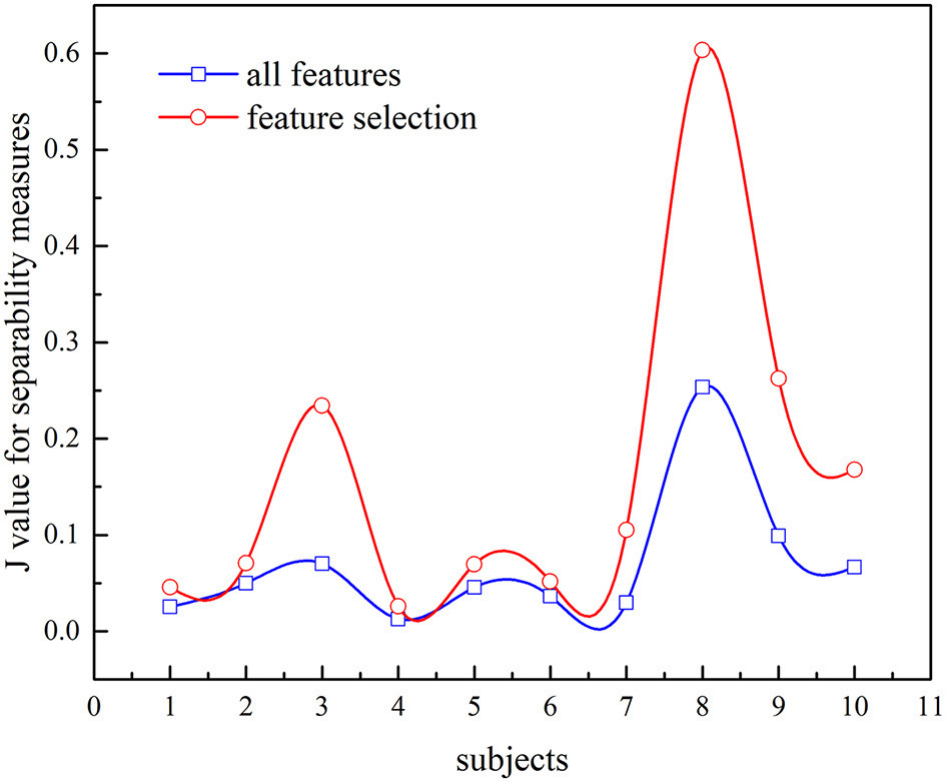
Comparison of *J*_*F*_ values between all features and feature selection for k-complex detection.

In this study, we also investigate the separability of the two different feature sets using *J*_*F*_. The larger the value of *J*_*F*_ is, the more separable the features are. Figure 10 presents the value of *J*_*F*_ and compares different feature sets (all features or selected features are used). It is evident that the *J*_*F*_ based on selected features is higher, which confirmed that the selected features can characterize the k-complex effectively. It can be confirmed by the inferences drawn from Figure 9. According to these results, the feature selection method was more effective, particularly in AUC, time comparison, and separability estimation. Furthermore, the experimental outcomes presented in Figures 9, 10 confirm that the feature selection method is more effective.

### 3.3. Performance for various classification models

**Figure 11:**
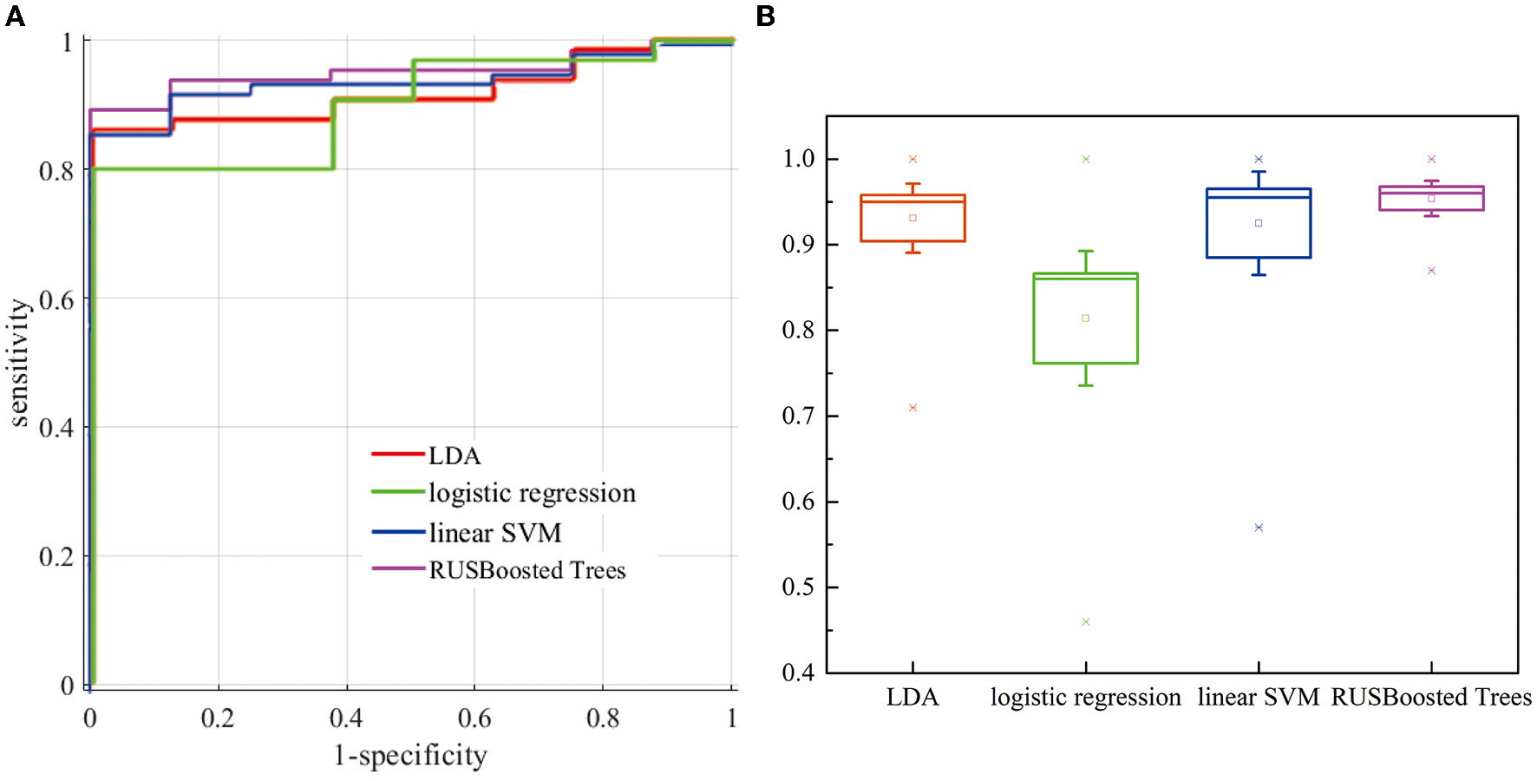
**(A)** Evaluation of the ROC curves (the plot of sensitivity vs. (1-specificity) for distinguishing k-complexes) for subjects 1. **(B)** Comparison of AUC for different methods.

For this research, we have verified several classification methods such as linear discriminant analysis (LDA), logistic regression, linear support vector machine (linear SVM), and RUSBoosted tree. Figure 11A indicates the receiver operating characteristic (ROC) curve for different classification methods. According to the results, the line in the upper left represents better performance in the detection of k-complexes. The area under the curve (AUC) of 1 indicates a perfect classification performance. Although this comparison is for the data set of subject 1, it has to be noticed that the k-complex classification can be improved using RUSBoosted tree methods. Figure 11B demonstrates a box plot of the area under the curve (AUC) for different pattern recognition methods. The AUC was obtained as 0.931 ± 0.085, 0.814 ± 0.166, 0.925 ± 0.127, and 0.954 ± 0.043 for LDA, logistic regression, linear SVM, and RUSBoosted tree, respectively. According to these results, we conclude that the AUC of the RUSBoosted tree is significantly better than others.

**Figure 12:**
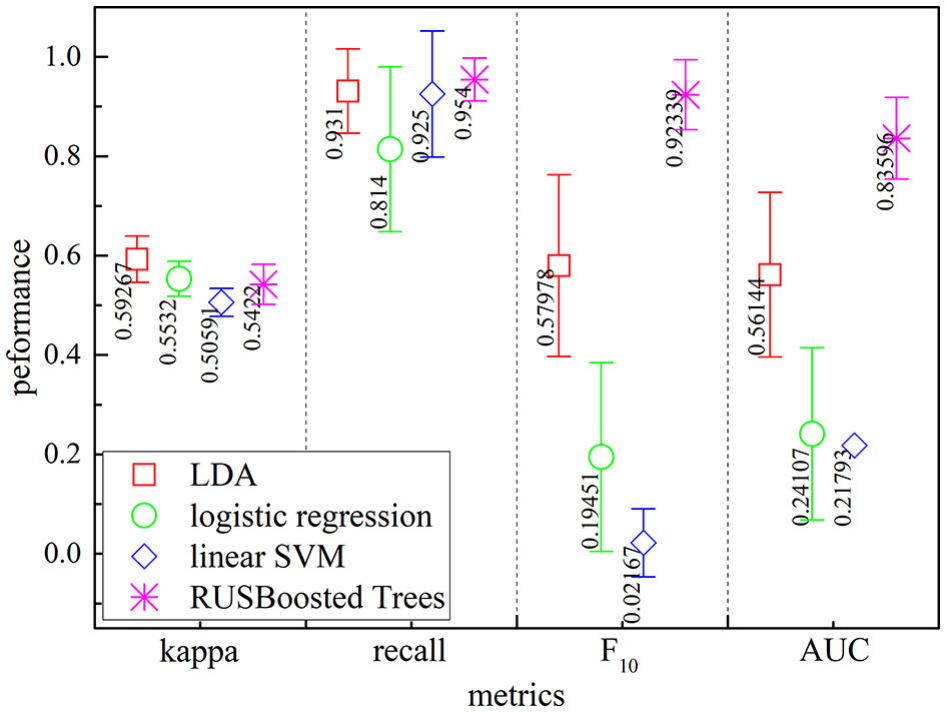
Performance comparison of the proposed method with different machine learning algorithms for the detection of k-complexes [LDA (red diamond), logistic regression (green circle), linear SVM (blue diamond), and RUSBoosted tree (pink star)]. Error bars correspond to the standard error of the mean.

The purpose of this investigation is to establish the suitability of the RUSBoosted tree algorithm for imbalanced dataset problems. The performance of the RUSBoosted tree algorithm is investigated for several traditionally state-of-the-art classifiers including LDA, logistic regression, and linear SVM. For further evaluation, Figure 12 reports the performance of some of these classifiers for the proposed scheme. The kappa coefficient, recall measure, AUC, and *F*_10_-score were used to evaluate the effectiveness of the proposed scheme. The proposed method achieved an average performance of recall measure, AUC, and *F*_10_-score of 92.34 ± 7.06%, 95.4 ± 4.32%, and 83.59 ± 8.23%, respectively. Depending on the results, the performance based on the kappa coefficient, recall measure, and *F*_10_-score provided evidence that the RUSBoosted tree surpassed other algorithms in the detection of the k-complexes. However, the performances based on the kappa coefficient using the RUSBoosted tree (54.22 ± 4.04%) are slightly worse than linear discriminant analysis (59.26 ± 14.67%). In summary, the prediction results confirmed a superiority value for different metrics and a balanced classification performance. It also indicated that the prediction algorithm based on the RUSBoosted tree model was tending to outperform than the traditional classifiers, especially for the minority classes.

### 3.4. Performance comparison of the proposed method based on the ratio of segment number

**Figure 13:**
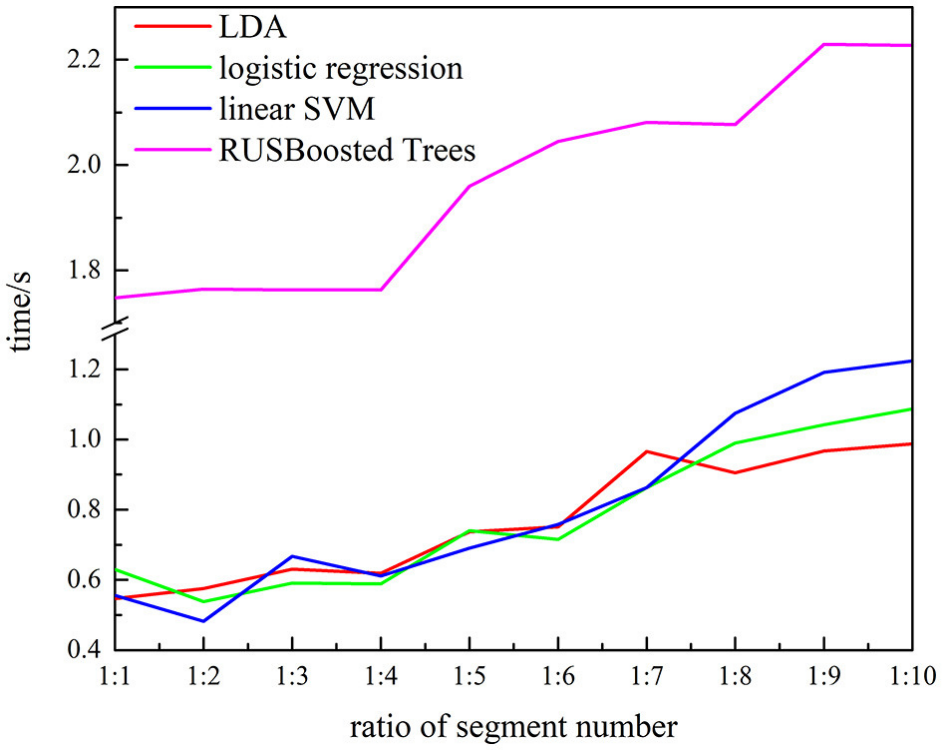
Relationship between the execution time and ratio of segment numbers for subject 1 (the number of k-complex is fixed as 263, and the segment number of non–k-complex is multiple of the number of k-complex from 1 to 10).

**Figure 14:**
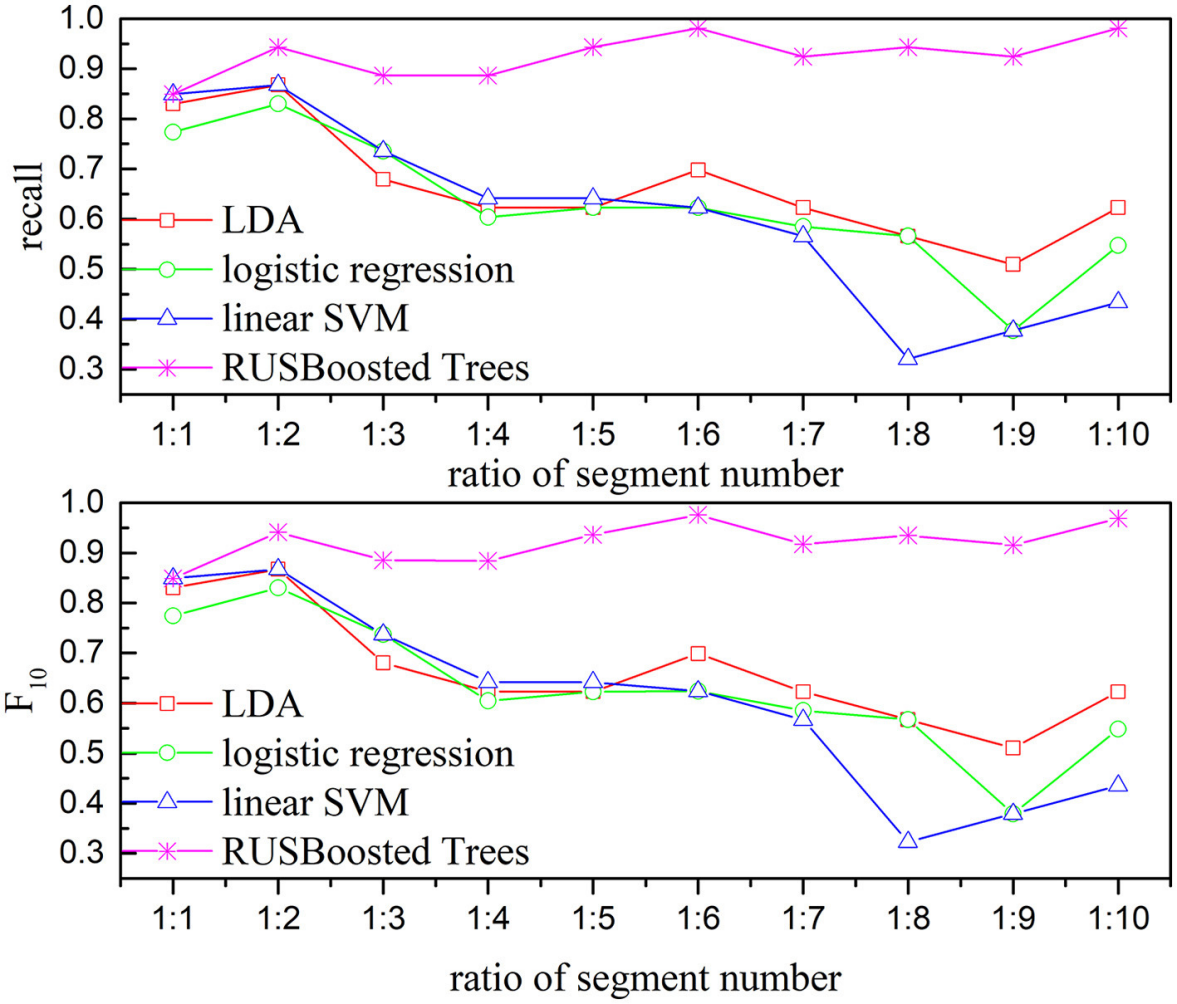
Relationship between the performance-based kappa and F_10_ and the ratio of segments number for subject 1 (the number of k-complex is fixed as 263, and the segment number of non–k-complex is multiple of the number of k-complex from 1 to 10).

To verify the performance of the proposed methods, the execution time, recall, and F_10_ scores are used. Figure 13 presents the execution time of the RUSBoosted tree model and the others classifiers. For further analysis, we assume that the number of the segments of the k-complex is fixed at 263, and the number of the segments of the non–k-complex is outnumbering k-complex (the number of segments of the non–k-complex increased from 1 to 10 times compared to the number of the segments of k-complex, and the number of segments was selected randomly from the database). The time to train the classification model was deemed as execution time. According to Figure 13, the slowest execution time was recorded with the RUSBoosted tree model compared with other classifiers. Along with the increasing number of segments, the execution time is also increased dramatically. In addition, the performance was also compared with the other three classifiers based on recall and F_10_ scores. Figure 14 achieves the results that the proposed method is slightly increased along with the increase in the ratio of the number of the segments between non–k-complex and k-complex. While the other classifiers' performance significantly decreased. High F_10_ values mean that the proposed method is inclined to small samples. From these results, we can get the conclusion that the proposed method was suitable to deal with the imbalanced dataset.

### 3.5. Comparison with existing methods based on Scenario 1

According to previously reported methods, some of the automatic k-complex detection methods have been estimated using the same database as discussed in Section 2.1. In Table 4, the proposed method is compared with existing methods. Krohne et al. (2014) detected k-complexes using wavelet transformation combined with feature thresholds with the same database. In this study, pseudo-k-complexes were identified from each EEG segment and then the feature threshold method was used to reject false positives. A mean recall of 74% was achieved. Parekh et al. (2015) reported their results of the k-complex detection using a fast non-linear optimization algorithm, an average recall and kappa of 61% and 0.54 were achieved, respectively. Another study was made by Ranjan et al. (2018), in which a fuzzy algorithm combined with an artificial neural network was used to detect k-complex, they reported an average accuracy and specificity of 87.65 and 76.2%, respectively. A fractal dimension coupled with an undirected graph features technique was utilized by Al-Salman et al. (2019b) to detect k-complexes. The accuracy and specificity of 97 and 94.7% were reported, and the performance was highest than others. Oliveira et al. (2020) focused on designing a multitaper-based k-complex detection method in EEG signals and achieved a recall of 85.1%. The proposed method outperforms the other methods in almost all performance metrics (accuracy and specificity), except the method of fractal dimension coupled with undirected graph features (Al-Salman et al., 2019b). In terms of recall and kappa, the proposed method achieves the highest performance. These results demonstrated that the proposed method achieved a better performance in terms of detection performance.

**Table 4:** Performance comparisons between the proposed method and other different detection methods with the same datasets based on Scenario 1.

**Methods**	**Accuracy (%)**	**Recall (%)**	**Specificity (%)**	**Kappa (%)**
Wavelet transformation (Krohne et al., 2014)	/	74	/	/
Spare optimization (Parekh et al., 2015)	/	61	/	54
Fuzzy neural network (Ranjan et al., 2018)	87.65	/	76.2	/
Short-term event extraction algorithm (Yazdani et al., 2018)	**/**	67.79	**/**	/
Fractal dimension coupled with undirected graph features (Al-Salman et al., 2019b)	**97**	/	**94.7**	/
Multitaper-based method (Oliveira et al., 2020)	/	85.1	/	/
Proposed methods	92.18	**92.41**	92.41	**54.54**

The bold value indicates the best performances are highlighted compared with other methods.

### 3.6. Comparison based on different scenarios

As already mentioned, some of the automatic k-complex detection methods have been proposed and compared with the proposed method with the regard to the scenarios previously discussed, as shown in Table 5. In Scenario 1, the proposed methods achieved a mean accuracy of 92.19 ± 3.9% and a mean recall of 92.41 ± 7.47%. The proposed method achieved a dramatically better recall than others (Devuyst et al., 2010; Yazdani et al., 2018; Oliveira et al., 2020), but slightly worse accuracy. A higher recall value indicates that the proposed method is able to detect the most of small samples (true k-complex marked by an expert).

**Table 5:** Performance comparisons between the proposed method and other existing methods for Scenarios 1 and 2.

**Methods**	**Scenario 1**		**Scenario 2**	
	**Accuracy (%)**	**Recall (%)**	**Accuracy (%)**	**Recall (%)**
Devuyst et al. (2010)	98.59	61.72	99.29	60.94
Yazdani et al. (2018)	**98.78**	67.79	**99.3**	73.02
Oliveira et al. (2020)	/	85.1 ± 5.05	/	77.2 ± 15.5
Proposed methods	92.19 ± 3.9	**92.41** **±7.47**	87.95 ± 6.16	**80.85** **±11.33**

The bold value indicates the best performances are highlighted compared with other methods.

In Scenario 2, compared to previous studies, the trade-off accuracy and recall obtained from the proposed method are similar to those obtained in Scenario 1. Compared to Scenario 1, the mean accuracy and recall are smaller, i.e., 87.95 ± 6.16% and 80.85 ± 11.33%, respectively. The reason why the recall and accuracy decrease for the scenario may be that the second expert marked few labels as k-complex compared to expert 1. It is consistent with Table 1. It is denoted that the proposed method was effective to detect the k-complex.

## 4. Conclusion

This study developed a k-complex detection scheme, consisting of TQWT, multi-domain features, feature selection, and RUSBoosted tree algorithm to overcome the shortages of the existing classification–misclassification of classifier training from the imbalanced data. According to the results, the highest recall value was achieved for the proposed scheme. The results denoted that the methods could be worth utilizing in the automatic identify the k-complex for sleep specialists. It has been evidenced that the proposed scheme is comparable to or better than the state-of-the-art classifiers. The results also show that the ability of the RUSBoosted tree model to deal with the imbalanced classification problems compared with the state-of-art methods is quite well. In general, according to the experimental outcomes, we can conclude that the proposed scheme can relieve physicians of the burden of visually inspecting a large volume of EEG data.

However, the study suffers from several drawbacks. First, it is necessary for researchers to locate the locations of the k-complex in the related epochs. Second, the proposed scheme relied on a single channel to detect k-complex. While as one of the important features of brain activity, the interaction between brain regions is not fully utilized.

## Data availability statement

Publicly available datasets were analyzed in this study. This data can be found at: https://zenodo.org/record/2650142.

## Ethics statement

Ethical review and approval was not required for the study on human participants in accordance with the local legislation and institutional requirements. Written informed consent for participation was not required for this study in accordance with the national legislation and the institutional requirements. Written informed consent was not obtained from the individual(s) for the publication of any potentially identifiable images or data included in this article.

## Author contributions

YL contributed to the conception and design of the study. YL and XD performed the statistical analysis and wrote the first draft of the manuscript. Both authors contributed to the manuscript revision and read and approved the submitted version.
